# The Rab18/Ras/ERK/FosB/MMP3 Signaling Pathway Mediates Cell Migration Regulation by 2′3′-cGAMP

**DOI:** 10.3390/ijms26125758

**Published:** 2025-06-16

**Authors:** Yu Deng, Runjie Yuan, Pengda Liu

**Affiliations:** 1 Lineberger Comprehensive Cancer Center, The University of North Carolina at Chapel Hill, Chapel Hill, NC 27599, USA; runjie_yuan@med.unc.edu; 2 Department of Biochemistry and Biophysics, The University of North Carolina at Chapel Hill, Chapel Hill, NC 27599, USA; 3 Department of Microbiology and Immunology, The University of North Carolina at Chapel Hill, Chapel Hill, NC 27599, USA

**Keywords:** 2′3′-cGAMP, MAPK, MMP3

## Abstract

The unique secondary messenger 2′3′-cGAMP, produced by cGAS in response to cytosolic dsDNA, plays a critical role in activating innate immunity by binding to and activating STING via cell-intrinsic, autocrine, or paracrine mechanisms. Recently, we identified Rab18 as a novel, STING-independent binder of 2′3′-cGAMP. Binding of 2′3′-cGAMP to Rab18 promotes Rab18 activation and induces cell migration. However, the downstream mechanisms by which 2′3′-cGAMP-induced Rab18 activation regulates cell migration remain largely unclear. Herein, using phospho-profiling analysis, we identify MAPK signaling as a key downstream effector of the 2′3′-cGAMP/Rab18 axis that promotes the expression of FosB2 and drives cell migration. Furthermore, we identify MMP3 as a major transcriptional target of FosB2, through which the 2′3′-cGAMP/Rab18/MAPK/FosB2 signaling pathway positively regulates cell migration. Together, our findings provide new mechanistic insights into how 2′3′-cGAMP signaling controls cell migration and suggest the potential of MAPK inhibitors to block 2′3′-cGAMP-induced migratory responses.

## 1. Introduction

Innate immunity is broadly present across most mammalian cell types and serves as the first line of defense against pathogens. The presence of cytosolic nucleic acids acts as a danger signal, triggering robust innate immune surveillance. Double-stranded DNA (dsDNA), when present in the cytoplasm, is detected by the DNA sensor cyclic GMP-AMP synthase (cGAS). Upon binding to dsDNA, cGAS undergoes conformational changes and catalyzes the conversion of ATP and GTP into the unique asymmetric secondary messenger 2′3′-cyclic GMP-AMP (2′3′-cGAMP) [1,2]. 2′3′-cGAMP binds to STING (stimulator of interferon genes) on the endoplasmic reticulum (ER), promoting STING trafficking to the Golgi apparatus. There, STING recruits TBK1, which phosphorylates IRF3, ultimately driving the transcription of type I interferons [3] that initiate innate and adaptive immune responses [4]. In addition to acting intracellularly, 2′3′-cGAMP can be exported to the extracellular space by the transporter ABCC1 [5] and taken up by neighboring cells through channels and transporters such as LRRC8C [6,7], SLC46A2 [8], SLC19A1 [9,10], and connexin 43 [11], facilitating immune activation in an autocrine or paracrine manner.

Beyond its role in innate immunity, 2′3′-cGAMP also interacts with other cellular proteins. For example, it binds to EF1A1 to inhibit protein translation [12] and, as we recently reported, Rab18, through which it enhances cell migration [13]. These findings suggest that 2′3′-cGAMP has broader cellular functions beyond its canonical immune signaling role. In our previous study, we demonstrated that 2′3′-cGAMP directly binds to Rab18, leading to its activation and subsequent induction of *FosB2* transcription. These findings identified Rab18 and FosB2 as two key signaling nodes through which 2′3′-cGAMP regulates cell migration. However, the mechanism by which Rab18 activation by 2′3′-cGAMP leads to *FosB2* transcription and how FosB2, in turn, modulates cell migration remains unclear. In this study, we aim to address these two questions to provide a more comprehensive understanding of the signaling pathway by which 2′3′-cGAMP controls cell migration.

## 2. Results

### 2.1. Phospho-Profiling Reveals That 2′3′-cGAMP Induces ERK Activation in a Rab18-Dependent Manner

**Figure 1:**
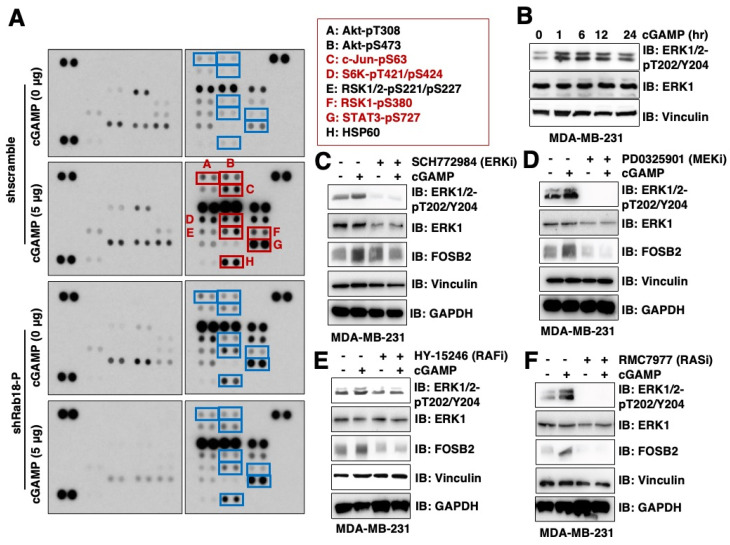
Phospho-profiling identifies MAPK as a critical kinase signaling downstream of 2′3′-cGAMP/Rab18. (**A**) Immunoblot (IB) analysis of whole-cell lysates (WCL) from the indicated MDA-MB-231 cells. Where indicated, indicated cells were treated with 5 μg/mL 2′3′-cGAMP for 2 h. Notably, the cells were cultured in sub-confluency in all assays in this study. Phosphorylation events increased by 2′3′-cGAMP in shScramble but not in shRab18 cells are highlighted with red boxes and labeled with letters (denoted in associated box). (**B**) IB analysis of WCL from MDA-MB-231 treated with 2.5 μg/mL 2′3′-cGAMP for the indicated time periods. (**C**–**F**) IB analysis of WCL from MDA-MB-231 cells co-treated with 2.5 μg 2′3′-cGAMP and the indicated inhibitors for 12 h prior to cell collection. Where indicated, SCH772984 (2 μM), PD-0325901 (1 μM), HY-15246 (1 μM), and RMC7977 (10 nM) were used.

Given that the activation of small GTPases has been shown to stimulate kinases for signal transduction (for example, Ras activation induces phosphorylation and activation of RAF kinases [14,15], while Rheb [16] or Rags [17] activation triggers mTORC1 activation), we hypothesize that 2′3′-cGAMP-induced Rab18 activation may similarly promote kinase activation to facilitate FOSB2 transcription. We continued using MDA-MB-231 cells, as previously reported [13], due to their expression of both Rab18 and FOSB2 (Figure S1A). To identify potential kinases involved, we conducted a phospho-profiling analysis using the Proteome Profiler Human Phospho-Kinase Array Kit to examine protein phosphorylation events induced by 2′3′-cGAMP in a Rab18-dependent manner in MDA-MB-231 cells (Figure 1A). Our results showed that 2′3′-cGAMP treatment induced several protein phosphorylation events in cells. When focusing on phosphorylation events specifically induced by 2′3′-cGAMP in shscramble but not shRab18 cells, we identified phosphorylation of Akt and several ERK downstream targets, including c-Jun-pS63 [18], S6K-pT421/pS424, RSK1/2-pS221/pS227, RSK1-pS380 [19], and STAT3-pS727 [20] (Figure 1A).

We next further validated Akt and ERK phosphorylation. We found that 2′3′-cGAMP slightly increased Akt-pS473 signals (Figure S1B) but more significantly induced ERK1/2-pT202/Y204 phosphorylation (Figure 1B). Notably, unlike growth factors and other canonical activators of the RAS/MEK/ERK pathway, 2′3′-cGAMP induced ERK activation in a relatively delayed manner, with significant activation observed only 60 min after 2′3′-cGAMP treatment (Figure S1C). These findings suggest that 2′3′-cGAMP-induced Rab18 activation likely leads to ERK kinase activation, therefore enhancing phosphorylation of ERK downstream targets.

### 2.2. 2′3′-cGAMP/Rab18-Induced ERK Activation Is Dependent on the RAS/RAF/MEK Pathway

To determine whether ERK activation mediates 2′3′-cGAMP/Rab18-induced FosB2 expression, we pretreated the cells with the ERK inhibitor SCH772984. SCH772984 effectively blocked ERK phosphorylation and suppressed 2′3′-cGAMP-induced FosB2 expression in both MDA-MB-231 (Figure 1C) and HeLa (Figure S1D) cells. These findings suggest that ERK activation is essential for 2′3′-cGAMP/Rab18-induced FosB2 expression. ERK activation is canonically triggered by the RAS/RAF/MEK signaling cascade. To investigate how 2′3′-cGAMP-induced Rab18 activation leads to ERK activation, we sequentially inhibited MEK1/2, RAF, and RAS. Inhibition of MEK1/2 with PD0325901 (Figure 1D), RAF with Tovorafenib (Figure 1E), or RAS with RMC7977 (Figure 1F) each significantly reduced FosB2 expression induced by 2′3′-cGAMP in MDA-MB-231 cells. Collectively, these results indicate that 2′3′-cGAMP-induced Rab18 activation promotes ERK signaling through the RAS/RAF/MEK/ERK kinase cascade.

### 2.3. The RAS/RAF/MEK/ERK Pathway Mediates 2′3′-cGAMP-Induced Cell Migration

**Figure 2:**
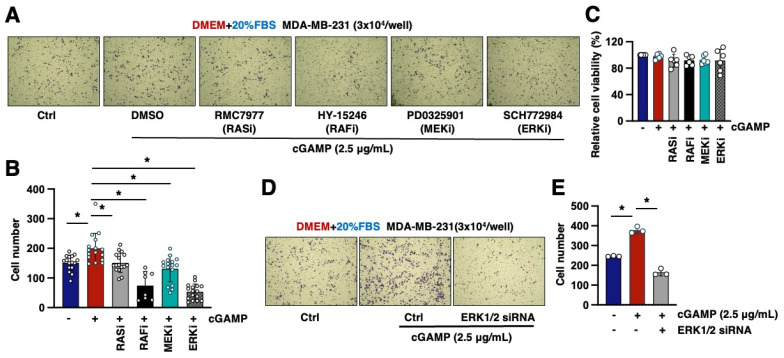
Pharmacological inhibition of MAPK signaling reduces 2′3′-cGAMP-induced cell migration. (**A**) Representative images from transwell assays of MDA-MB-231 cells under indicated conditions for 24 h. Where indicated, the indicated inhibitor was co-administrated with 2.5 μg/mL 2′3′-cGAMP for 24 h. SCH772984 (2 μM), PD-0325901 (1 μM), HY-15246 (1 μM), and RMC7977 (10 nM). (**B**) Quantification of the number of cells in transwell assays from (**A**). Error bars were calculated as the mean ± SD; n number of replicates are as indicated in each panel. * *p* < 0.05 (one-way ANOVA test). (**C**) MTT assays of MDA-MB-231 cells under the indicated conditions for 24 h. Where indicated, the indicated inhibitor was co-administrated with 2.5 μg/mL 2′3′-cGAMP for 24 h. SCH772984 (ERKi, 2 μM), PD-0325901 (MEKi, 1 μM), HY-15246 (RAFi, 1 μM), and RMC7977 (RASi, 10 nM). (**D**) Representative images from transwell assays of MDA-MB-231 cells under the indicated conditions for 24 h. Where indicated, MDA-MB-231 cells were transfected with 100 nM Erk (1/2) siRNA for 72 h and then harvested for co-administration with 2.5 μg/mL 2′3′-cGAMP for 24 h. (**E**) Quantification of the number of cells in transwell assays from (**D**). Error bars were calculated as the mean ± SD; n biological replicates are as indicated in each panel. * *p* < 0.05 (one-way ANOVA test).

Previously, we reported that 2′3′-cGAMP facilitates cell migration through binding and activating Rab18/FosB signaling [13]. Given that we found activation of RAS/RAF/MEK/ERK signaling was indispensable for 2′3′-cGAMP-induced FosB2 expression (Figure 1), we next examined whether RAS/RAF/MEK/ERK signaling mediates 2′3′-cGAMP-induced cell migration. To this end, we pre-treated MDA-MB-231 cells with DMSO or inhibitors targeting RAS (RMC7977), RAF (HY-15246), MEK (PD0325901), or ERK (SCH772984), respectively, prior to 2′3′-cGAMP stimulation. In contrast with 2′3′-cGAMP treatment that induced MDA-MB-231 cell migration, blocking each of the kinases reduced cell migration (Figure 2A,B). Notably, these inhibitor treatments did not cause any apparent defects in cell proliferation (Figure 2C). To address the concern that pharmacological ERK inhibition might block 2′3′-cGAMP-induced cell migration through off-target effects, we also used siRNAs to deplete endogenous ERK1/2 (Figure S1E). Consistently, genetic ERK1/2 knockdown similarly impaired 2′3′-cGAMP-induced migration in MDA-MB-231 cells (Figure 2D,E). Together, these results suggest that RAS/RAF/MEK/ERK signaling is not only necessary for 2′3′-cGAMP-induced FosB2 expression, but also indispensable for 2′3′-cGAMP-induced cell migration.

### 2.4. MMPs Are Key Transcriptional Targets of FosB2 That Mediate Cell Migration Induced by 2′3′-cGAMP

**Figure 3:**
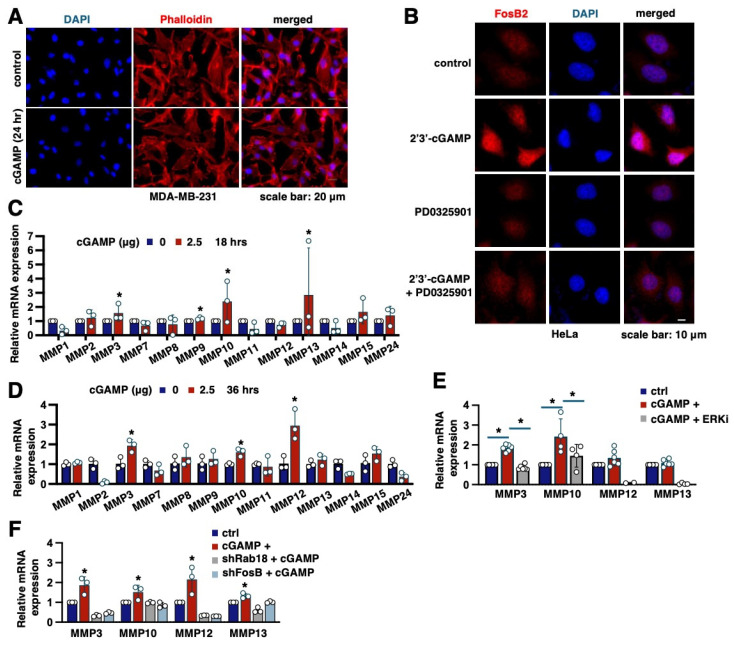
MMP3 is a potential transcriptional target of the 2′3′-cGAMP/Rab18/MAPK/FosB2 signaling axis. (**A**) Representative immunofluorescent (IF) images of indicated MDA-MB-231 cells with DAPI and phalloidin staining. Where indicated, cells were treated with 2.5 μg/mL 2′3′-cGAMP for 24 h. (**B**) Representative IF images of indicated HeLa cells. Where indicated, HeLa cells were treated with 2.5 μg/mL 2′3′-cGAMP alone, PD-0325901 (1 μM) alone, or in combination for 24 h. (**C**,**D**) RT-PCR analysis of mRNA expression of indicated MMPs from MDA-MB-231 cells treated with 2.5 μg/mL 2′3′-cGAMP for either 18 h (**C**) or 36 h (**D**). Error bars were calculated as the mean ± SD; n biological replicates are as indicated in each panel. * *p* < 0.05 (one-way ANOVA test). (**E**) RT-PCR analysis of mRNA expression of indicated MMPs from MDA-MB-231 cells treated with 2.5 μg/mL 2′3′-cGAMP alone or in combination with SCH772984 (2 μM) for 12 h. Error bars were calculated as the mean ± SD; n biological replicates are as indicated in each panel. * *p* < 0.05 (one-way ANOVA test). (**F**) RT-PCR analysis of mRNA expression of indicated MMPs from indicated MDA-MB-231 cells treated with 2.5 μg/mL 2′3′-cGAMP for 18 h. Error bars were calculated as the mean ± SD; n biological replicates are as indicated in each panel. * *p* < 0.05 (one-way ANOVA test).

Notably, 2′3′-cGAMP induced migration of MDA-MB-231 cells with minimal alteration of the cytoskeletal architecture, as indicated by phalloidin staining of actin filaments (Figure 3A). In addition, 2′3′-cGAMP treatment increased FosB2 cellular abundance, while MEK inhibition by PD0325901 blocked this process (Figure 3B), further confirming a critical role of ERK in governing FosB2 expression and function. Given that FosB2 is a member of the FOS family of transcription factors, which form AP-1 (activating protein-1) complexes with members of the Jun family [21], we next sought to identify transcriptional targets regulated by FosB2 that might modulate 2′3′-cGAMP-induced cell migration. Notably, previous studies have shown that during trophoblast invasion (a critical process in human placentation), increased Fos expression enhances the transcription of matrix metalloproteinases (MMPs), facilitating invasion [22]. We thus examined the transcriptional response of a panel of MMPs, including MMP1, MMP2, MMP3, MMP7, MMP8, MMP9, MMP10, MMP11, MMP12, MMP13, MMP14, MMP15, and MMP24, following 2′3′-cGAMP treatment at two time points associated with cell migration: 18 h (Figure 3C) and 36 h (Figure 3D). Among them, MMP3 and MMP10 emerged as consistent hits at both time points, suggesting that they may be transcriptional targets of 2′3′-cGAMP-induced FosB2 (Figure 3C,D). However, ERK inhibition by SCH772984 only suppressed the 2′3′-cGAMP-induced upregulation of MMP3 but not MMP10 mRNA (Figure 3E). Furthermore, knockdown of either Rab18 or FosB2 reduced MMP3 transcription, but not MMP10, in the presence of 2′3′-cGAMP (Figure 3F). Together, these findings support a possible signaling cascade composed of 2′3′-cGAMP/Rab18/ERK/FosB2/MMP3.

### 2.5. MMP3 Is a Key Downstream Effector to Mediate 2′3′-cGAMP-Induced Cell Migration

**Figure 4:**
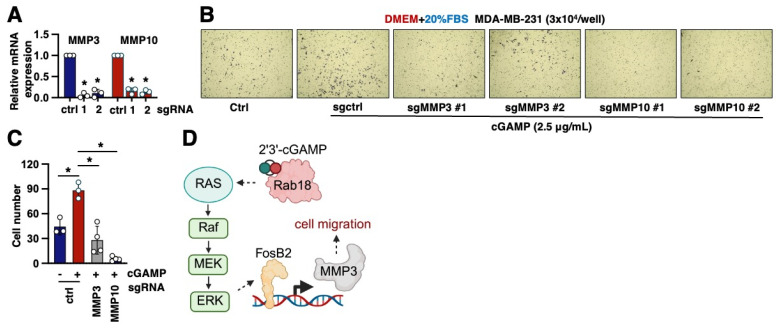
MMP3 depletion reduces 2′3′-cGAMP-induced cell migration. (**A**) RT-PCR analysis of MMP3 or MMP10 mRNAs in the indicated MDA-MB-231 cells. Error bars were calculated as the mean ± SD; n number of replicates are as indicated in each panel. * *p* < 0.05 (one-way ANOVA test). (**B**) Representative images from transwell assays of indicated MDA-MB-231 cells under the indicated conditions for 24 h. Where indicated, the cells were treated with 2.5 μg/mL 2′3′-cGAMP for 24 h. (**C**) Quantification of the number of cells in transwell assays from (**B**). Error bars were calculated as the mean ± SD; n number of replicates are as indicated in each panel. * *p* < 0.05 (one-way ANOVA test). (**D**) A cartoon illustration of the proposed model. Our data suggests a model where 2′3′-cGAMP binds to and activates Rab18, which in turn triggers the Ras/Raf/MEK/ERK MAPK signaling cascade, leading to the transcriptional activation of FosB2. FosB2 then enhances the expression of migration-promoting genes, including MMP3. Through this pathway, the 2′3′-cGAMP/Rab18/MAPK/FosB2/MMP3 signaling axis regulates cell migration.

To further assess the role of MMP3 in 2′3′-cGAMP-induced cell migration, we designed two independent sgRNAs targeting either MMP3 or MMP10, both of which effectively reduced their respective gene expression in MDA-MB-231 cells (Figure 4A). Notably, knockdown of either MMP3 or MMP10 significantly impaired 2′3′-cGAMP-induced cell migration (Figure 4B,C). However, since MMP10 expression appears to be regulated independently of the Rab18/FosB2 axis (Figure 3E,F), these findings suggest that MMP3, but not MMP10, functions as a critical downstream effector of the 2′3′-cGAMP/Rab18/ERK/FosB2 signaling pathway in promoting cell migration (Figure 4D). 

## 3. Discussion

Innate immunity plays a central role in eliminating infections by eradicating infected cells and activating the adaptive immune response. Beyond pathogen defense, it also contributes to various human diseases. Hyperactivation of innate immune pathways can lead to autoimmune disorders [23,24], whereas suppression of innate immunity is often hijacked by cancer cells to evade immune surveillance to facilitate tumorigenesis [25,26] or treatment resistance [27]. Consequently, strategies to enhance tumor-intrinsic innate immune signaling have been explored as cancer therapies. For example, 2′3′-cGAMP has been shown to enhance the efficacy of immune checkpoint blockade (ICB) in murine tumor models, primarily by activating STING/IFN-β signaling and promoting immune cell infiltration [28]. In parallel, pharmacological agonists targeting key components of the cytosolic DNA sensing pathway, such as cGAS and STING, have demonstrated efficacy in enhancing ICB responses in cancer treatment [29]. These findings support the idea that activation of the cGAS/cGAMP/STING pathway can generate a tumor-rejecting immune microenvironment.

In addition to its canonical role in STING activation, 2′3′-cGAMP has been shown to exert STING-independent biological effects. For example, it binds to and inhibits EF1A1, thereby suppressing protein translation [12]. Given the reliance of cell proliferation on active protein synthesis, this finding may suggest a potential anti-proliferative role of 2′3′-cGAMP. Furthermore, our previous work revealed that 2′3′-cGAMP directly binds and activates Rab18, a lipid droplet-localized small GTPase, promoting cell migration [13]. This raises a potential concern regarding the dual nature of 2′3′-cGAMP in cancer therapy: while it enhances antitumor immunity, it may also promote pro-metastatic cellular behaviors through cell-intrinsic mechanisms.

In the present study, we expand on our previous findings by dissecting the downstream signaling events of the 2′3′-cGAMP/Rab18 axis in regulating cell migration. Phosphoproteomic profiling identified activation of MAPK signaling, particularly the Ras/Raf/MEK/ERK cascade, as a downstream effector of Rab18 activation. This pathway leads to FosB2 transcription and expression. Moreover, we demonstrate that FosB2 directly promotes the transcription of MMP3, a key mediator of cell migration. Together, our data define a novel cell-intrinsic signaling axis composed of 2′3′-cGAMP/Rab18/MAPK/FosB2/MMP3 that regulates cell motility. In addition to our prior findings on targeting Rab18 prenylation with statins, this study highlights MAPK signaling components as potential therapeutic targets to mitigate the pro-migratory side effects of 2′3′-cGAMP, thereby improving the safety profile of cGAS/STING agonists as cancer immunotherapeutics.

Despite these insights, several mechanistic questions remain unresolved. It is unclear how Rab18 activation, presumably occurring on lipid droplets, leads to activation of Ras, which is primarily localized at the plasma membrane. Although lipid droplet-localized Ras-related proteins, such as Rab2A, have been reported [30], it remains unclear whether Ras could localize to lipid droplets. In addition, we cannot exclude the possibility that 2′3′-cGAMP/Rab18 activation generates diffusible signals that indirectly activate Ras at the membrane. Furthermore, the mechanism by which ERK signaling regulates FosB2 transcription remains to be elucidated, particularly the identity of the ERK-responsive transcription factor(s) involved. While we establish MMP3 as a major downstream effector of FosB2 in promoting cell migration, it remains to be determined whether MMP3 acts through its known metalloprotease activity in remodeling the extracellular matrix [31] or via alternative mechanisms. Notably, although the transwell inserts used in our studies are made of polyethylene terephthalate (PET), a material not susceptible to degradation by MMP3, their surfaces are tissue culture (TC)-treated. This treatment typically involves coating with extracellular matrix (ECM) proteins such as collagen, laminin, and fibronectin to enhance cell attachment. Therefore, it is plausible that MMP3 facilitates cell migration by remodeling these ECM components, thereby enabling cells to traverse the transwell pores. These questions merit further investigation to fully understand the biological implications of 2′3′-cGAMP signaling.

## 4. Materials and Methods

### 4.1. Cell Culture and Transfection

Human adenocarcinoma cell lines HeLa and MDA-MB-231 and human immortalized kidney cell lines HEK293 and HEK293T were cultured in DMEM medium supplemented with 10% FBS, 100 units of *penicillin,* and 100 mg/mL *streptomycin*.

Cell transfection was carried out using Lipofectamine 3000 or polyethylenimine (PEI) as previously described [13,32]. Lentiviral packaging for shRNA or cDNA expression and subsequent cell infections were performed following established protocols [33,34]. After infection, cells were selected in culture media with hygromycin (200 μg/mL) or puromycin (1 μg/mL), depending on the viral vector used, for at least 72 h to eliminate non-infected cells.

### 4.2. Reagents

For small-molecule inhibitors, the reagents used in this study are listed as follows: RMC-7977 (RAS inhibitor) (MedChemExpress (MCE), Monmouth junction, NJ, USA HY-156498); Tovorafenib (pan-RAF inhibitor) (MedChemExpress HY-15246); Mirdametinib or PD0325901 (MEK1/2 inhibitor) (MedChemExpress HY-10254); and SCH772984 (ERK1/2 inhibitor) (MedChemExpress HY-50846). 

### 4.3. Antibodies

The antibodies used in this study are listed as follows: anti-FOSB2 antibody (Cell Signaling Technology 2251S, Danvers, MA, USA); anti-phospho-ERK1/2 (Thr202/Tyr204) antibody (Cell Signaling Technology 4370S); anti-ERK1 antibody (Santa Cruz Biotechnology sc-271269, Dallas, TX, USA); anti-Rab18 antibody (Santa Cruz Biotechnology sc-393169); anti-Vinculin antibody (Santa Cruz Biotechnology sc-25336); anti-GAPDH antibody (Proteintech 60004-1-Ig, Rosemont, IL, USA); anti-rabbit immunoglobulin G (IgG); horseradish peroxidase (HRP)-linked antibody (Cell Signaling Technology 7074); anti-mouse IgG; and HRP-linked antibody (Cell Signaling Technology 7076).

### 4.4. Primers

The primers used in RT-PCR are listed as follows:

MMP1-RT-F: ATGAAGCAGCCCAGATGTGGAG

MMP1-RT-R: TGGTCCACATCTGCTCTTGGCA

MMP2-RT-F: AGCGAGTGGATGCCGCCTTTAA

MMP2-RT-R: CATTCCAGGCATCTGCGATGAG

MMP3-RT-F: CACTCACAGACCTGACTCGGTT

MMP3-RT-R: AAGCAGGATCACAGTTGGCTGG

MMP24-RT-F: CCAGTACATGGAGACGCACAAC

MMP24-RT-R: TCCTCTCCGATGGTGAGTGGAT

MMP7-RT-F: TCGGAGGAGATGCTCACTTCGA

MMP7-RT-R: GGATCAGAGGAATGTCCCATACC

MMP8-RT-F: CAACCTACTGGACCAAGCACAC

MMP8-RT-R: TGTAGCTGAGGATGCCTTCTCC

MMP9-RT-F: GCCACTACTGTGCCTTTGAGTC

MMP9-RT-R: CCCTCAGAGAATCGCCAGTACT

MMP10-RT-F: TCCAGGCTGTATGAAGGAGAGG

MMP10-RT-R: GGTAGGCATGAGCCAAACTGTG

MMP11-RT-F: GAGAAGACGGACCTCACCTACA

MMP11-RT-R: CTCAGTAAAGGTGAGTGGCGTC

MMP12-RT-F: GATGCTGTCACTACCGTGGGAA

MMP12-RT-R: CAATGCCAGATGGCAAGGTTGG

MMP13-RT-F: CCTTGATGCCATTACCAGTCTCC

MMP13-RT-R: AAACAGCTCCGCATCAACCTGC

MMP14-RT-F: CCTTGGACTGTCAGGAATGAGG

MMP14-RT-R: TTCTCCGTGTCCATCCACTGGT

MMP15-RT-F: CTGGCTCTTTCGAGAAGCGAAC

MMP15-RT-R: TCTCCTCGTTGAAGCGCCAGTA

### 4.5. siRNA, shRNAs and sgRNAs

p44/p42 (Erk1/2) MAP kinase siRNA was purchased from Cell Signaling Technology (#6560, Danvers, MA, USA).

shRNA plasmids were constructed by inserting synthesized shRNAs into pLKO-puro or pLKO-blast vectors. shRab18-P (TRCN0000021979), shRab18-Q (TRCN0000021983), and shRab18-R (TRCN0000021981) constructs were purchased from Sigma-Aldrich (St. Louis, MO, USA).

The shRNA primers for FosB are listed as follows:

shFosB-1: GCCGAGTCTCAATATCTGTCTC

shFosB-2: GCCAACCACAATTCAATGAAT

sgRNA plasmids were constructed by inserting synthesized sgRNAs into the lentiCRISPRv2-puro vector or the lentiCRISPRv2-blast vector.

The sgRNAs used in this study are listed below:

MMP3-sg1-F: CACCGGTACGAGCTGGATACCCAAG

MMP3-sg1-R: AAACCTTGGGTATCCAGCTCGTACC

MMP3-sg2-F: CACCGCTTGCTAGTAACTTCATATG

MMP3-sg2-R: AAACCATATGAAGTTACTAGCAAGC

MMP10-sg1-F: CACCGTTGCAGCGGACAAATACTGG

MMP10-sg1-R: AAACCCAGTATTTGTCCGCTGCAAC

MMP10-sg2-F: CACCGACATTGCTAGGCGAGATAGG

MMP10-sg2-R: AAACCCTATCTCGCCTAGCAATGTC

### 4.6. RT-PCR Quantifications

Total RNA from MDA-MB-231 or HeLa cells was isolated using the EZ-10 DNAaway RNA miniprep kit (Bio Basic BS88136, Markham, ON, Canada), reverse-transcribed into cDNA using the iScript reverse transcription supermix (Bio-Rad 1708840, Hercules, CA, USA), and mixed with 2XSYBR Green universal master mix (Thermo Fisher 4309155, Waltham, MA, USA). Raw sample CT was obtained with the QuantStudio 6 Flex real-time PCR system and converted to ΔCt by subtracting the CT value of the house-keeping gene U6. Relative gene expression was calculated using the equation below: ΔΔCt = ΔCt (treated sample) − ΔCt (control sample). Fold changes between the experimental group and the control group were calculated using the equation below: Fold change = 2^(−ΔΔCt).

### 4.7. Transwell Assays

A total of 30,000 live MDA-MB-231 cells, mixed with or without RAS/RAF/MEK/ERK inhibitors, were seeded into 8.0 µm Falcon permeable support inserts (CORNING 353097, NY, USA) in 0.3 mL of serum-free DMEM medium (Gibco 11995-065, Miami, FL, USA) with or without 2.5 μg/mL cGAMP. The inserts were placed on top of 0.6 mL of DEME media supplemented with 20% fetal bovine serum, together in a 24-well tissue culture plate. The cells were incubated at 37 °C for 24 h, before being washed with 1× Dulbecco’s phosphate-buffered saline (DPBS) 3 times and fixed with 4% paraformaldehyde for 10 min. Inserts with fixed cells were then washed again with DPBS 3 times and stained in 0.4% crystal violet blue for 30 min. Residual stains were washed off with DPBS-3 times and air-dried at room temperature for 30 min. Cells that failed to cross the membrane were removed with a Q-tip, and positively stained cells were quantified using the EVOS XL Core imaging system (Thermo Scientific, Waltham, MA, USA).

### 4.8. Immunoblot Analyses

For immunoblotting analyses, cells were lysed in EBC buffer (50 mM Tris, pH 7.5, 120 mM NaCl, 0.5% NP-40) or RIPA buffer (50 mM Tris, pH 7.5, 150 mM NaCl, 1% Triton X-100, 1% sodium deoxycholate, 0.1% SDS), both supplemented with protease inhibitor cocktails and phosphatase inhibitor cocktails. The protein concentrations of the whole-cell lysates were determined using the Bio-Rad Bradford protein assay and measured with a NanoDrop OneC (Thermo Scientific, Waltham, MA, USA). Equal amounts of total proteins in whole-cell lysates were resolved via SDS-PAGE and subjected to immunoblotting with the indicated antibodies.

### 4.9. Immunofluorescence Assays

Cells grown on glass coverslips were fixed with 4% paraformaldehyde in 1× PBS for 20 min at room temperature, followed by permeabilization with 0.2% Triton X-100 for 20 min. After washing, the cells were incubated in blocking buffer (5% bovine serum albumin and 0.1% Triton X-100 in PBS) for 1 h at room temperature. Primary antibodies were applied and incubated overnight at 4 °C, followed by incubation with secondary antibodies for 1 h at room temperature. Coverslips were mounted using ProLong Gold Antifade Reagent, and fluorescent signals were visualized using a Keyence BZ-X700 fluorescence microscope (Itasca, IL, USA).

### 4.10. MTT Cell Viability Assay

During this stage, 3000 MDA-MB-231 cells were seeded in each well of 96-well plates for MTT assays to monitor cell viability at the indicated time periods. Briefly, at indicated time points post-cell seeding, 10 μL MTT solution was added into each well and incubated in the culture incubator (37 °C with 5% CO_2_) for 4 h. Then, the medium was removed and 100 μL DMSO was added into each well to dissolve the formazan crystal and incubated for 10 min at 37 °C. After thorough mixing, absorbance at 540 nm was measured using the BioTek Cytation 5 Cell Imaging reader (Agilent, Santa Clara, CA, USA).

### 4.11. Statistical Analysis

Statistical analyses were performed using GraphPad Prism 8 software. A *p*-value of ≤0.05 was considered statistically significant. Data are presented as the mean ± SD from at least two or three independent experiments, as indicated in the figure legends. Differences between control and experimental groups were assessed using a t-test, one-way ANOVA, or two-way ANOVA, as appropriate.

## Data Availability

All supporting data are provided in the supplemental information section and can be requested from the corresponding authors.

## Supplementary Materials

The following supporting information can be downloaded at: https://www.mdpi.com/article/10.3390/ijms26125758/s1.

## Abbreviations

The following abbreviations are used in this manuscript:

MAPK	mitogen-activated protein kinase
FosB2	FosB proto-oncogene
MMP	matrix metalloproteinase
RAS	rat sarcoma virus
RAF	RAF protein-serine/threonine kinase
MEK	mitogen-activated protein kinase kinase
ERK	extracellular signal-regulated kinase
ICB	immune checkpoint blockade
STING	stimulator of interferon genes
cGAS	cyclic GAMP synthase
