# Supplementary material for: The Rab18/Ras/ERK/FosB/MMP3 Signaling Pathway Mediates Cell Migration Regulation by 2′3′-cGAMP
Source: Int J Mol Sci. 2025 Jun 16;26(12):5758. doi: 10.3390/ijms26125758 (PMC12192691; doi:10.3390/ijms26125758)
Supplement: Supplementary file 1 [file ijms-26-05758-s001.zip › ijms-3667724-supplementary.pdf]

The Rab18/Ras/ERK/FosB/MMP3 signaling pathway mediates cell migration regulation by 2'3'-cGAMP

Yu Deng, Runjie Yuan, and Pengda Liu

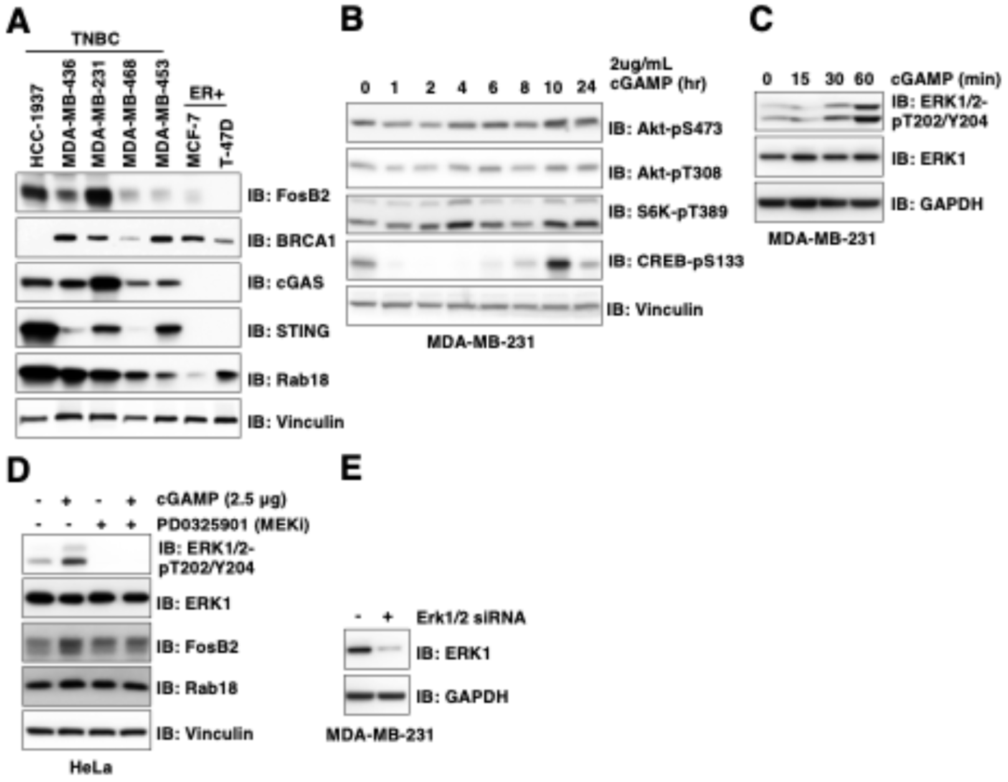

Figure S1. 2’3’-cGAMP binding to Rab18 activates MAPK signaling in cells.

(A) IB analysis of WCL from indicated cell lines.

(B) IB analysis of WCL from MDA-MB-231 cells treated with 2 μg/mL 2’3’-cGAMP for indicated time periods.

(C) IB analysis of WCL from indicated MDA-MB-231 cells. Where indicated, indicated cells were treated with 2.5 μg/mL 2’3’-cGAMP for 0, 15, 30, 60 min.

(D) IB analysis of WCL from HeLa cells treated with 2.5 μg/mL 2’3’-cGAMP or/and PD0325901 (1 μM) for 12 hours prior to cell collection.

(E) IB analysis of WCL from MDA-MB-231 cells transfected with 100 nM SgnalSilence p44/42 MAPK (ERK1/2) siRNA for 72 hours.
